# Molecularly Imprinted Deoxynivalenol Surface Plasmon Resonance Sensor Based on Sulfur-Doped Boron Graphitic Carbon Nitride

**DOI:** 10.3390/foods15030481

**Published:** 2026-01-30

**Authors:** Müge Mavioğlu Kaya, Haci Ahmet Deveci, Bahar Bankoğlu Yola, İlknur Polat, Sena Bekerecioğlu, Necip Atar, Mehmet Lütfi Yola

**Affiliations:** 1 Department of Molecular Biology and Genetic, Faculty of Art and Science, Kafkas University, Kars 36000, Türkiye; m.mavioglu@kafkas.edu.tr; 2 Department of Nutrition and Dietetics, Faculty of Health Sciences, Gaziantep University, Gaziantep 27000, Türkiye; h_ahmet_deveci@gantep.edu.tr; 3 Department of Engineering Basic Sciences, Faculty of Engineering and Natural Sciences, Gaziantep Islam Science and Technology University, Gaziantep 27260, Türkiye; bahar.bankogluyola@gibtu.edu.tr; 4 Department of Nutrition and Dietetics, Faculty of Health Sciences, Hasan Kalyoncu University, Gaziantep 27010, Türkiye; ilknur.polat@hku.edu.tr (İ.P.); sena.bekerecioglu@hku.edu.tr (S.B.); 5 Department of Chemical Engineering, Faculty of Engineering, Pamukkale University, Denizli 20160, Türkiye; natar@pau.edu.tr; 6 Department of Biology, Faculty of Science, Ankara University, Ankara 06100, Türkiye; 7 Integrated Technologies Research Center (BUTAM), Ankara University, Ankara 06690, Türkiye

**Keywords:** deoxynivalenol, surface plasmon resonance, nanocomposite, graphitic carbon nitride, molecularly imprinting polymers

## Abstract

Deoxynivalenol (DEOX), a dangerous mycotoxin, causes serious health problems for humans and animals. Hence, the on-site monitoring of DEOX has begun to be important in the health and food sectors in recent years. In the present study, a molecularly imprinted surface plasmon resonance (SPR) sensor based on sulfur-doped boron graphitic carbon nitride (S-B-g-C_3_N_4_) was developed and applied for detecting DEOX in drinking water and orange juice samples, achieving high recovery. After the S-B-g-C_3_N_4_ nanocomposite was synthesized via thermal polycondensation and microwave treatment with a highly environmentally friendly approach, a SPR chip was modified with the S-B-g-C_3_N_4_ nanocomposite considering the high affinity between gold and sulfur. Then, the molecularly imprinted SPR sensor based on the S-B-g-C_3_N_4_ nanocomposite was prepared in the presence of methacryloylamidoglutamic acid (MAGA) as the monomer and N,N′-azobisisobutyronitrile (AIBN) as the initiator. The DEOX-imprinted SPR sensor based on the S-B-g-C_3_N_4_ nanocomposite showed linearity from 1.0 to 10.0 ng L^−1^, with a limit of quantification (LOQ) of 1.0 ng L^−1^ and a limit of detection (LOD) of 0.30 ng L^−1^. Finally, the selectivity, repeatability, and reproducibility of the DEOX-imprinted SPR sensor based on the S-B-g-C_3_N_4_ nanocomposite were investigated.

## 1. Introduction

The toxic compounds produced by certain molds are called mycotoxins. Numerous mycotoxins, such as zearalenone (ZEN), DEOX, and aflatoxin B1 (AFB1), exist in nature. These compounds are commonly present in many foods due to processing under inappropriate humidity and temperature conditions [1,2]. DEOX, a type of mycotoxin, is produced by molds of the *Fusarium* genus, particularly *Fusarium graminearum* and *Fusarium culmorum*. Chemically, it is a secondary metabolite with a trichothecene group, it is resistant to heat, and it is not completely removed during processing or cooking [3]. DEOX, with its 12,13-epoxide ring and a double bond at C9-10, inhibits cellular protein synthesis, causing ribotoxic stress. Therefore, it is also known as “ribotoxin” and is thought to exert its toxic effects through this mechanism of action [4]. 

In addition, DEOX is one of the most important mycotoxins in terms of agricultural processes, the food industry, and the food chain. It is widely found in grains such as wheat, barley, rye, rice, and corn [5] and is found in more than half of the grain products in Europe, North America, and Asia [6]. The prevalence of DEOX in food is considered a serious problem. The European Food Safety Authority (EFSA) has set maximum permissible levels of DEOX in animal feed products and various foodstuffs [7]. Additionally, the provisional maximum tolerable daily intake (PMTDI) of DEOX and its acetylated forms was set to 1 μg/kg body weight/day by the Food and Agriculture Organization (FAO)/WHO Expert Committee on Food Additives (JECFA) [8]. DEOX’s contamination of agricultural products and its global prevalence pose potential health threats and have serious toxics effect on humans when taken into the body [9]. The primary signs of food poisoning are diarrhea, nausea, and vomiting [3]. Chronic exposure to DEOX has been reported to cause feeding refusal, reproductive dysfunction, immune suppression, weight loss, and neuronal anomalies in adults, while it causes growth retardation in children [7,10]. In addition, it causes cytotoxicity and genotoxicity [11] and damages intestinal epithelial tissue [12]. 

Because DEOX is frequently found in food products and poses a threat to human health, its detection is of paramount importance. The accurate detection of DEOX contributes to the protection of public health by preventing contaminated products from entering the food chain. ELISA (Enzyme-Linked Immunosorbent Assay), HPLC (High-Pressure Liquid Chromatography), GC-MS (Gas Chromatography–Mass Spectrometry), and LC-MS/MS (Liquid Chromatography–Mass Spectrometry) are some methods available for detecting DOX, according to the literature [13,14,15,16]. The use of all of these analytical methods is limited due to the difficulty of sample preparation, long analysis times, high costs, and the need for specialized personnel. Recently, more economical, faster, and more sensitive analytical methods have been developed for the detection of DEOX [17]. Among these analytical methods, SPR is an optical analysis method that excites the free electrons located on a metal surface via polarized light emitted at a certain angle. The binding of analyte molecules to the sensor surface causes a change in the surface’s refractive index, which is detected as a shift in the resonance angle. Thus, SPR measures specific interactions between biomolecules in real time [18].

Metal-free nanomaterials such as g-C_3_N_4_ are superior catalysts due to their mechanical, physical, and chemical stabilities, as well as low cost, in comparison with those of metal-based nanomaterials [19,20]. g-C_3_N_4_ is an important nanomaterial because of its high stability and low toxicity [21,22,23]. In addition, it has a wide tunable bandgap, affecting its crystallinity and morphological structure. Nonetheless, its limited activity is an important problem owing to the need for fast charge recombination. Also, hexagonal boron nitride (h-BN) possesses high mechanical stability and thermal conductivity, acting as a strong absorber under difficult conditions [24]. Additionally, it has high bandgap energy, indicating suitability for catalytic applications. h-BN can be doped with heteroatoms to increase its surface area and charge migration, producing more active sites. Hence, doping with boron and nitrogen changes the functions of some nanomaterials. Boron graphitic carbon nitride (B-g-C_3_N_4_) is a multi-layered nanomaterial having properties similar to those graphite, with an interplanar distance of 0.350 nm. In the structure, boron and nitrogen are in the same positions as carbon [25]. B-g-C_3_N_4_ is a nanomaterial can possesses the superior functions of both boron carbide and boron nitride [26,27], and, thus, novel nanocomposites can be developed that avoid the problems experienced with its individual components. The multi-doping treatment of heteroatoms can increase their catalytic functions, ensuring high stability. Nonmetals such as boron and sulfur can be doped via interstitial or substitutional doping. However, the metal doping of g-C_3_N_4_ does not substitute the carbon and nitrogen atoms [28]. In the literature, heteroatoms including oxygen and sulfur were doped onto carbon nanomaterials by using trithiocyanuric acid, producing approximately twelve times more hydrogen than graphitic carbon nitride [29]. In addition, the oxygen reaction was completed by using triple doping treatment instead of mono- and dual-doping treatments. Synergy between catalytic activity and reaction kinetics was obtained via doping heteroatoms [30]. Doping treatment with sulfur can decrease hexagonal boron nitride’s bandgap, and sulfur 3d orbitals can provide intermediate states, providing the energy for electron transition [31].

Molecular imprinting is a three-step synthesis process involving the polymerization of monomers in the presence of a target molecule to form artificial polymers containing specific recognition sites. The molecularly imprinted polymers (MIPs) produced via this process contain specific binding sites that are suitable for the shape, size, and functional groups of the target molecule when the target molecule is removed [32]. The mechanism of MIPs is based on the interaction of these specific cavities with the analyte, with the cavities demonstrating high selectivity and binding affinity for the analyte. MIPs are widely used in food analysis because they are cheap, durable, and highly stable [33]. In the literature, MIP-based sensors were developed for DEOX detection. First, some MIPs were prepared for DEOX analysis by using 2-trifluoromethylacrylic acid as the functional monomer and ethylene glycol dimethacrylate (EGDMA) as the cross-linking agent. According to HPLC experiments, MIPs enable the highly selective analysis of DEOX enrichment [34]. Secondly, a molecularly imprinted polypyrrole-based SPR sensor was developed, and it showed linearity from 1.0 to 100.0 ng mL^−1^ and an LOD of 1.0 ng mL^−1^ [35]. Finally, an impedimetric sensor for DEOX detection based on MIPs and a gold electrode was developed using an o-phenylenediamine monomer. The sensor showed linearity from 5.0 to 500.0 ng mL^−1^ and an LOD of 0.3 ng mL^−1^ in food samples [36].

This study aimed to develop a method for the sensitive and selective determination of DEOX mycotoxins based on a molecularly imprinted polymer and sulfur-doped boron graphitic carbon nitride. To achieve this aim, sulfur-doped boron graphitic carbon nitride nanocomposite was prepared by using thermal polycondensation and microwave treatment, producing an environmentally friendly sensor. Then, a molecularly imprinted polymer decorated with S-B-g-C_3_N_4_ was prepared and used as an SPR surface material for DEOX detection for the first time. Thus, this SPR sensor can be used to avoid the crucial health problems resulting from mycotoxin exposure by enabling the easy identification of DEOX.

## 2. Materials and Methods

### 2.1. Chemicals and Apparatus

**Scheme 1:**
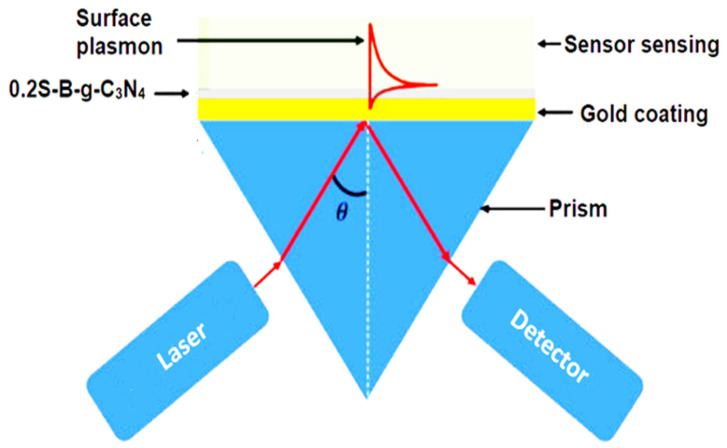
SPR system layout in this study in Kretschmann configuration.

DEOX, ochratoxin (OCH), AFB1, ZEA, aflatoxin B2 (AFB2), h-BN, melamine (MEL), boric acid (H_3_BO_3_), MAGA, EGDMA, 2-hydroxyethylmethacrylate (HEMA), AIBN, phosphate buffer (PB), and sodium chloride (NaCl) were bought by Sigma-Aldrich Merck Group (Darmstadt, Germany). SPR system (GenOptics, SPRi-Lab, Orsay, France) was used for analytical applications (Scheme 1), and the other apparatuses used for structural analysis are presented in the Supplementary Materials.

In this study, raw data were often noisy or contained deviations originating from the device. Before starting the analysis, the following steps were applied:

- Zeroing: the moment the injection started was determined as the starting point on the time axis (t = 0) and the signal axis (y = 0).
- Reference subtraction: to eliminate bulk effects and non-specific bindings that occurred during the experiment, data from an empty channel without a solution were removed from the master data.
- Cropping: parts irrelevant to the analysis, such as during stabilization, were removed from the dataset.

The following procedures were applied for response extraction:

- Association: the analyte interacted with the sensor surface, and an increase in response unit (RU) was observed.
- Dissociation: the analyte flow was interrupted, and the separation of bound molecules was observed.
- Maximum response (Rmax): the response value at the point where the sensor surface reached saturation was determined.

### 2.2. Preparation of g-C_3_N_4_, B-g-C_3_N_4_, and S-B-g-C_3_N_4_ Nanocomposites

g-C_3_N_4_ was produced by using direct thermal polycondensation of MEL according to the protocol described in our study [37].

For B-g-C_3_N_4_ synthesis, a solution including MEL (1.50 g) and H_3_BO_3_ (1.00 g) in pure water (100.0 mL) was prepared and stirred at 100 °C. After the dilution of the MEL-H_3_BO_3_ complex (3.0 g) in pure water (20.0 mL), it was subjected to ultrasound waves for 20 min. Then, the suspension was subjected to microwaving at 400 W for 30 min, and B-g-C_3_N_4_ was washed with pure water and dried at 50 °C.

S-B-g-C_3_N_4_ nanocomposites were prepared by mixing B-g-C_3_N_4_ (1.0 g) with different ratios of sulfur powder (0.2%, 0.4%, and 0.6%). The mixtures were put in a ball-milling tool for 4 h. The obtained products were labeled 0.2S-B-g-C_3_N_4_, 0.4S-B-g-C_3_N_4_, and 0.6S-B-g-C_3_N_4_ nanocomposites.

### 2.3. Modification of SPR Chip with 0.2S-B-g-C_3_N_4_ and Preparation of DEOX-Imprinted SPR Sensor with 0.2S-B-g-C_3_N_4_

Before SPR measurements, the gold-coated SPR chips were cleaned using acidic piranha solution (10.0 mL, 3:1 H_2_SO_4_:H_2_O_2_, *v*/*v*) at 25 °C. Gold-coated SPR chips were placed in a container containing acidic piranha solution and left in a shaking bath for 15 min. After drying in atmospheric nitrogen, the modification process was completed due to the high affinity between gold and sulfur. For this purpose, 0.2S-B-g-C_3_N_4_ (2.0 mg mL^−1^) was dropped onto the surface of clean SPR chips at 25 °C and left to stand for 30 min to allow self-assembly (0.2S-B-g-C_3_N_4_/SPR) [38].

After the MAGA:DEOX complex was prepared in pH 6.0 PB (3.0 mL) in a 2:1 molar stoichiometric ratio, a solution containing AIBN (2.0 mg), HEMA (3.0 mL), and EGDMA as the cross-linking agent (3.0 mL) was added to the MAGA:DEOX complex solution, which served as the polymerization solution. After the polymerization solution (2.0 mL) was dropped onto the 0.2S-B-g-C_3_N_4_/SPR chip surface, the spin-coating technique was used to ensure a homogeneous and monolayer distribution of the solution on the chip surface. The substrate was rotated at a speed of 5000 rpm for 10 s at 25 °C. The SPR chips were then left under ultraviolet (UV) light at an intensity of 1 mW cm^2-^ for 1 h, and the polymerization process proceeded. The prepared SPR surface was called MIP/0.2S-B-g-C_3_N_4_/SPR. In molecular imprinting sensor technology, NIP sensors are prepared by molecular imprinting without the target molecule to demonstrate the advantage of MIPs in highly selectively recognizing the target molecule. This process is called as the molecular imprinting effect. A DEOX-free polymerization solution was prepared, and NIP SPR surfaces (NIP/0.2S-B-g-C_3_N_4_/SPR) were prepared using the technique described above to examine the effect of molecular imprinting.

### 2.4. DEOX Removal from MIP/0.2S-B-g-C_3_N_4_/SPR Sensor and Analysis Process

To break the strong electrostatic interactions between DEOX and MAGA, an aqueous solution of NaCl (2.0 mol L^−1^) was used as the desorption solution in this study. For this purpose, the prepared MIP/0.2S-B-g-C_3_N_4_/SPR chips were immersed in vessels containing NaCl (2.0 mol L^−1^, 10.0 mL) solution to remove DEOX molecules from the chip surface for a desorption time of 10 min. The MIP/0.2S-B-g-C_3_N_4_/SPR sensor surface with the eluted DEOX was dried under a vacuum at 25 °C.

The analysis procedure began with PB (5.0 mL, pH 6.0) as the equilibration solution with a 1.0 mL min^−1^ flow rate through the SPR cell channel for 10 min. Then, different concentrations of the prepared DEOX solutions (1.0–10.0 ng L^−1^) were sequentially delivered to the SPR chip surface (at a 1.0 mL min^−1^ flow rate). After the system reached a plateau for 40 min, the regeneration process was initiated with NaCl (2.0 mol L^−1^, 10.0 mL) at a 1.0 mL min^−1^ flow rate for 10 min, and the adsorption–desorption–regeneration cycle was completed.

### 2.5. Sample Preparation

After the homogeneous drinking water and homogeneous orange juice samples (10.0 mL) were separately put into volumetric containers (100.0 mL), a centrifugation process was first performed for 5 min to remove contaminants that might be present in the drinking water and orange juice samples. Since no potential compounds affecting the results were found, the developed sensor was directly applied with the drinking water and orange juice samples. Then, the drinking water and orange juice samples were diluted with pH 6.0 PB (2.0 mol L^−1^), and the samples were transferred to an SPR cell at a 1.0 mL min^−1^ flow rate. The analysis was performed at 25 °C.

## 3. Results and Discussion

### 3.1. Characterization of S-B-g-C_3_N_4_ Nanocomposite

**Figure 1:**
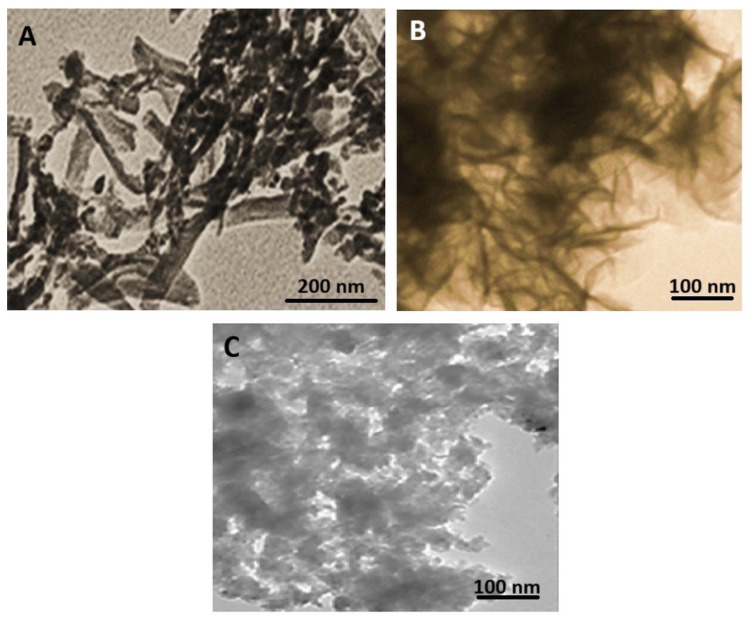
TEM images of (**A**) g-C_3_N_4_, (**B**) B-g-C_3_N_4_, and (**C**) 0.2S-B-g-C_3_N_4_ nanocomposites.

Transmission electron microscopy (TEM) images were obtained for investigating the surface morphology (Figure 1). The unique, uniform, and flexible nanofibers with a diameter of 25–40 nm suggested the successful preparation of g-C_3_N_4_ that had a aggregated, layered, wafer-like morphology (Figure 1A). The agglomerated surface structure containing boron, which was in the form of a pile, confirmed the formation of B-g-C_3_N_4_ (Figure 1B). Then, the TEM image of the 0.2S-B-g-C_3_N_4_ nanocomposite revealed a more agglomerated surface structure, including large numbers of nanometer-scale in-plane holes with a hierarchical mesoporous structure, suggesting the incorporation of sulfur into B-g-C_3_N_4_ (Figure 1C) [39]. In addition, the high porosity ensured the high catalytic ability of the 0.2S-B-g-C_3_N_4_ nanocomposite via mass transfer. Finally, the presence of sulfur, boron, carbon, and nitrogen in the energy-dispersive X-ray (EDX) spectroscopy results indicated the successful preparation of the 0.2S-B-g-C_3_N_4_ nanocomposite (Figure S1).

The X-ray diffraction (XRD) patterns of g-C_3_N_4_, h-BN, and B-g-C_3_N_4_ are presented in Figure S2A. The XRD peak at 27.05°, corresponding to the (002) plane, suggested the conjugated g-C_3_N_4_ had a stacking form [40,41]. The XRD pattern belonging to h-BN showed peaks at 27.26° and 42.07° (weak), attributed to the (002) and (100) planes, verifying the incorporation of graphitic material [42,43,44]. The XRD pattern of B-g-C_3_N_4_ demonstrated the specific diffraction peak of h-BN, corresponding to the (002) plane [39], and the small peak at 11.16° was attributed to the (100) plane in triazine g-C_3_N_4_ [40]. The XRD patterns (Figure S2B) of B-g-C_3_N_4_, 0.2S-B-g-C_3_N_4_, 0.4S-B-g-C_3_N_4_, and 0.6S-B-g-C_3_N_4_ nanocomposites are given. The peaks specific to g-C_3_N_4_ at 27.05° and 13.49° were observed in the XRD patterns of the B-g-C_3_N_4_, 0.2S-B-g-C_3_N_4_, 0.4S-B-g-C_3_N_4_, and 0.6S-B-g-C_3_N_4_ nanocomposites. However, the XRD peak intensities at 27.05° and 13.49° decreased after sulfur doping owing to the formation of highly exfoliated B-g-C_3_N_4_ nanosheets and the reduced planar size of the B-g-C_3_N_4_ nanosheets [45]. Crystallite size was also calculated by Scherrer’s equation, and the crystal sizes were found to be 7.69 nm, 5.07 nm, and 3.47 nm for 0.2S-B-g-C_3_N_4_, 0.4S-B-g-C_3_N_4_, and 0.6S-B-g-C_3_N_4_. These results collectively confirmed that bi-nonmetallic doping with sulfur resulted in thinner particles and caused exfoliation.

**Figure 2:**
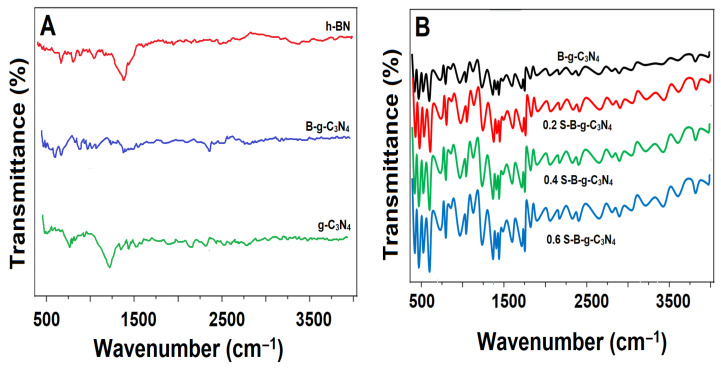
FTIR spectra of (**A**) g-C_3_N_4_, B-g-C_3_N_4_, and h-BN; (**B**) B-g-C_3_N_4_ and S-B-g-C_3_N_4_ nanocomposites.

The Fourier-transform infrared spectroscopy (FTIR) spectra of the g-C_3_N_4_, B-g-C_3_N_4_, h-BN, and S-B-g-C_3_N_4_ nanocomposites are presented in Figure 2A,B. g-C_3_N_4_ showed obvious absorption bands in the range of 1300–1600 cm^−1^, suggesting the vibration of tri-s-triazine structures. The absorption band at 1570 cm^−1^ was attributed to –C=N– groups, and the band at 2385 cm^−1^, related to –CΞN–, corresponded to the cyano groups formed from acetonitrile. Moreover, the absorption bands at 3200–3400 cm^−1^ were related to –NH_2_ and –NH amine groups and –OH groups. The absorption band at 811 cm^−1^ was attributed to the bending vibration of triazine rings [22,40]. The FTIR spectrum of h-BN revealed two obvious absorption bands: one at 808 cm^−1^, corresponding to the out-of-plane vibration of –B–N–B– groups, and another at 1373 cm^−1^. attributed to the in-plane stretching of the B–N bonds. The absorption bands in the range of 3075–3250 cm^−1^ corresponded to the presence of –NH_2_ and –OH groups [39]. The FTIR spectrum of B-g-C_3_N_4_ contained absorption bands at 810 cm^−1^ and 1417 cm^−1^, corresponding to –B–N–B– stretching and B–N vibration, respectively [46]. A wide absorption band between 3192 cm^−1^ and 3381 cm^−1^ was attributed to–NH and –OH vibrations, and the band at 2347 cm^−1^ corresponded to the –CΞN– group. Finally, the presence of –C–N heterocycles was indicated by obvious bands at 1230–1590 cm^−1^ (Figure 2A) [47]. Thus, these absorption bands, which were found to be consistent with those in the literature, indicated the successful synthesis of B-g-C_3_N_4_.

The absorption band at 808 cm^−1^ was attributed to–B–N–B–out-of-plane vibration, and the absorption bands at 1230–1580 cm^−1^ corresponded to –C–N heterocycles in the B-g-C_3_N_4_ and S-B-g-C_3_N_4_ nanocomposites. In addition, the absorption band at 1384 cm^−1^ corresponded to –B–N stretching. However, the overlapping of –B–N and –C–N produced a minor change in the absorption bands, resulting from interlayer interactions [45]. Lastly, the broad absorption bands at 3120–3385 cm^−1^ corresponded to the overlapping of –N–H and –OH stretching (Figure 2B) [39], indicating the successful synthesis of the S-B-g-C_3_N_4_ nanocomposites. No new band was observed, indicating that bi-nonmetallic doping did not change the original g-C_3_N_4_ structure, which was in accordance with the XRD results.

The optical properties of the g-C_3_N_4_, h-BN and B-g-C_3_N_4_, and S-B-g-C_3_N_4_ nanocomposites were investigated using ultraviolet–visible (UV–Vis) diffuse reflectance (Figure S3). g-C_3_N_4_ had an obvious signal in the visible area, with an absorption band at 460 nm owing to π–π* or n–π* electronic transitions, belonging to the aromatic s-triazine ring [40]. h-BN and B-g-C_3_N_4_ showed absorption at 210 nm and 250 nm, respectively (Figure S3A). The optical properties were also revealed for the S-B-g-C_3_N_4_ nanocomposites (Figure S3B). The higher absorption band and the lower absorption band were attributed to –B–N and –C–N, respectively. Obvious shifts in the absorption edges were observed in the UV–Vis diffuse reflectance of the S-B-g-C_3_N_4_ nanocomposites, and this red shift increased with increasing sulfur content. Thus, the sulfur 3d orbitals formed intermediate states, suggesting a decrease in the energy required for photoelectron transition and a reduced bandgap. In conclusion, the S-B-g-C_3_N_4_ nanocomposites showed strong absorption in the visible light region, which was due to the generated localized electronic state caused by heteroatom doping in the bandgap.

**Figure 3:**
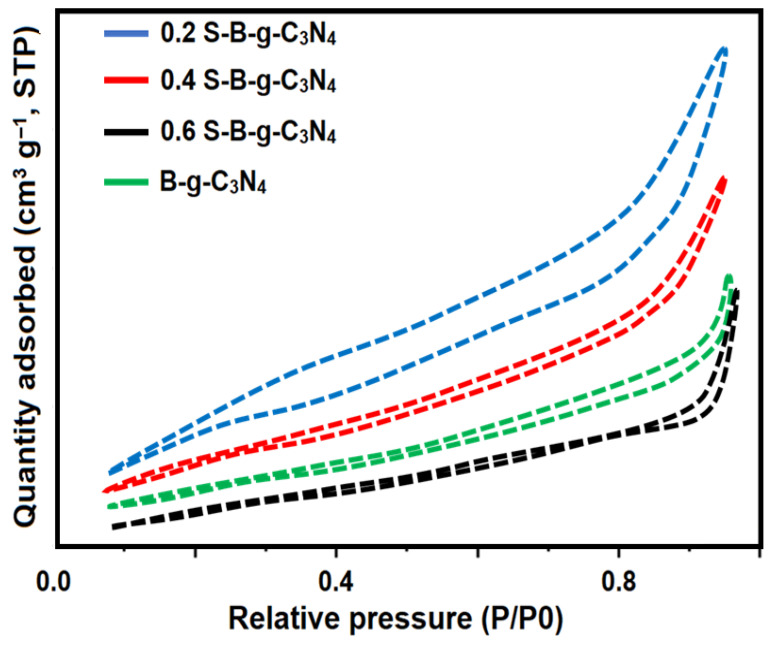
N_2_ adsorption–desorption isotherms of B-g-C_3_N_4_ and S-B-g-C_3_N_4_ nanocomposites.

N_2_ adsorption–desorption isotherms of B-g-C_3_N_4_ and S-B-g-C_3_N_4_ nanocomposites are presented in Figure 3. All samples isotherms were comparable, which were type IV isotherms with H3 hysteresis loops, confirming a mesoporous nature. The isotherms of the 0.2S-B-g-C_3_N_4_, 0.4S-B-g-C_3_N_4_, and 0.6S-B-g-C_3_N_4_ nanocomposites had greater nitrogen adsorption at higher relative pressures in comparison with B-g-C_3_N_4_ because of sulfur doping. The BET (Brunauer–Emmett–Teller) specific surface areas of the B-g-C_3_N_4_, 0.2S-B-g-C_3_N_4_, 0.4S-B-g-C_3_N_4_, and 0.6S-B-g-C_3_N_4_ nanocomposites were calculated as 50.41 m^2^ g^−1^, 157.13 m^2^ g^−1^, 76.43 m^2^ g^−1^, and 72.19 m^2^ g^−1^, respectively. The decreases in BET values with increasing sulfur content were due to the contraction of the B-g-C_3_N_4_ nano-layers with increasing sulfur content. It was found that bi-nonmetallic doping of the g-C_3_N_4_ structure increased the surface area and pore volume. The increases in specific surface area and pore volume could effectively promote the kinetics of the catalytic reaction by facilitating mass transfer.

### 3.2. FTIR and Atomic Force Microscopy (AFM) Studies of DEOX-Imprinted Film on 0.2S-B-g-C_3_N_4_/SPR Chip

According to the FTIR spectrum of DEOX-imprinted film on 0.2S-B-g-C_3_N_4_/SPR (Figure S4A), –OH stretching belonging to MAGA-HEMA, –CH stretching, carboxyl-carbonyl stretching, and –CO–O– stretching were observed at 3594 cm^−1^, 3004 cm^−1^, 1748 cm^−1^, and 1444 cm^−1^, respectively. These absorption modes confirmed the successful production of the DEOX-imprinted film on the 0.2S-B-g-C_3_N_4_/SPR chip, which is in agreement with the literature [38]. In addition, Figure S4B,C confirm the smooth SPR surface and dense polymeric layers on the SPR surfaces, respectively. Surface thicknesses of 1.83 ± 0.03 nm and 17.15 ± 0.01 nm were observed for the bare SPR and the DEOX-imprinted film on 0.2S-B-g-C_3_N_4_/SPR chips, indicating a thicker DEOX-imprinted film formed on 0.2S-B-g-C_3_N_4_/SPR chips.

### 3.3. pH Effect and Nanocomposite Effect on SPR

**Figure 4:**
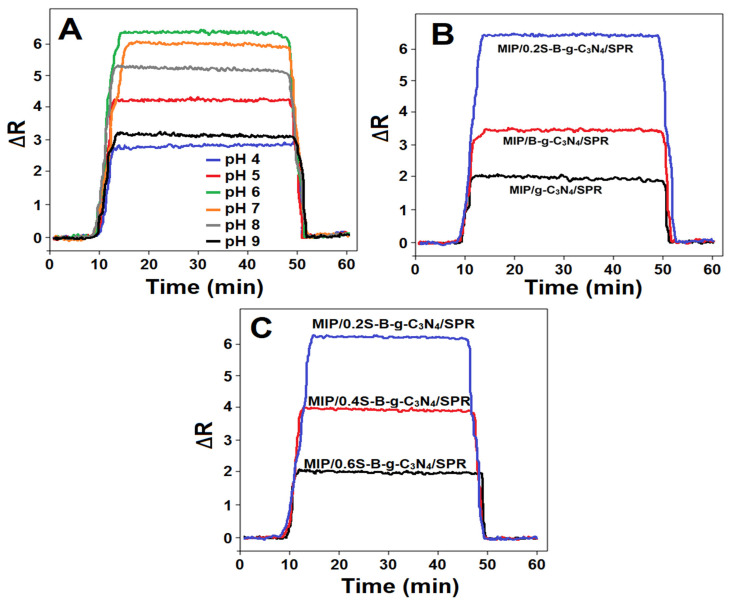
(**A**) SPR sensorgrams of MIP/0.2S-B-g-C_3_N_4_/SPR chips using PB with different pH values including 10.0 ng L^−1^ DEOX, (**B**) SPR sensorgrams of MIP/0.2S-B-g-C_3_N_4_/SPR, MIP/B-g-C_3_N_4_/SPR, and MIP/g-C_3_N_4_/SPR chips with 10.0 ng L^−1^ DEOX and (**C**) SPR sensorgrams of MIP/0.2S-B-g-C_3_N_4_/SPR, MIP/0.4S-B-g-C_3_N_4_/SPR, and MIP/0.6S-B-g-C_3_N_4_/SPR chips with 10.0 ng L^−1^ DEOX.

When analyzing food using sensors such as SPR, minimal changes in ambient pH significantly impact sensor sensitivity. Therefore, in this study, the results were first obtained under optimal conditions by optimizing pH. Because the MAGA monomer in the developed SPR sensor was acidic (carboxylic), MAGA:analyte interactions increased as the pH increased due to the ionization of the MAGA monomer. At more basic pH values, the MAGA:analyte affinity was lower because of the electrostatic repulsion due to the ionization of the analyte molecule. Thus, 6.0 was selected as the optimum pH value [48] (Figure 4A).

Figure 4B shows the effect of different nanocomposite modifications on the prepared SPR sensor. The sensor responses of the MIP/0.2S-B-g-C_3_N_4_/SPR, MIP/B-g-C_3_N_4_/SPR, and MIP/g-C_3_N_4_/SPR chips were examined. As expected, first, more stable SPR signals were obtained with MIP/0.2S-B-g-C_3_N_4_/SPR and MIP/B-g-C_3_N_4_/SPR chips due to the higher number of active regions on their surfaces due to boron doping in the presence of 10.0 ng L^−1^ DEOX in comparison with the MIP/g-C_3_N_4_/SPR chip. Then, the SPR signals of MIP/0.2S-B-g-C_3_N_4_/SPR chips were more stable and stronger due to the sulfur–gold affinity created via sulfur doping. The SPR signals were enhanced because of the self-assembled monolayers that formed via strong sulfur–gold–nitrogen covalent bonds [49]. Finally, Figure 4C demonstrates the effect of different sulfur contents in the S-B-g-C_3_N_4_ nanocomposite on the SPR sensor signals. In alignment with the BET analysis, the strongest SPR signals were obtained with MIP/0.2S-B-g-C_3_N_4_/SPR chips.

Scanning electron microscopy (SEM) images were recorded to evaluate the morphological differences between the MIP and NIP SPR chips, which had more-porous SPR surfaces (Figure S5A) and non-porous/smooth SPR surfaces (Figure S5B). As expected, the larger number of porous structures specific to the target molecule, DEOX, which were produced by molecular imprinting technology, were observed.

### 3.4. Sensitivity of MIP/0.2S-B-g-C_3_N_4_/SPR Chip

**Figure 5:**
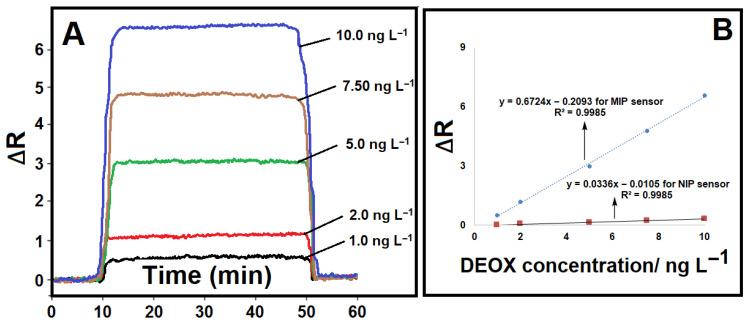
(**A**) SPR sensorgrams for different DEOX concentrations with MIP/0.2S-B-g-C_3_N_4_/SPR sensor in the presence of pH 6.0 PB (from 1.0 ng L^−1^ to 10.0 ng L^−1^ DEOX), (**B**) calibration curve of DEOX concentrations against the observed SPR signals for MIP/0.2S-B-g-CN_4_/SPR and NIP/0.2S-B-g-C_3_N_4_/SPR sensors.

In this study, the SPR method used has some advantages such as enabling real-time monitoring, requiring small sample volumes, and having a low detection limit in the nanogram range [50]. Hence, the obtained SPR signals (Figure 5A) showed linearity for 1.0–10.0 ng L^−1^ DEOX with MIP/0.2S-B-g-C_3_N_4_/SPR chips and had a calibration equation of y (ΔR) = 0.6724x (C_DEOX_, ng L^−1^) − 0.2093 (Figure 5B). In addition, linearity from 1.0 to 10.0 ng L^−1^ DEOX with a calibration equation of y (ΔR) = 0.0336x (C_DEOX_, ng L^−1^) − 0.0105 was obtained for NIP/0.2S-B-g-C_3_N_4_/SPR chips (Figure 5B). In comparison with MIP/0.2S-B-g-C_3_N_4_/SPR and NIP/0.2S-B-g-C_3_N_4_/SPR chips, the proposed MIP/0.2S-B-g-C_3_N_4_/SPR sensor detected DEOX with higher selectivity at all concentrations of DEOX due to its high imprinting selectivity. In addition, an LOQ of 1.0 ng L^−1^ and an LOD of 0.30 ng L^−1^ were obtained (related equations in Supplementary Materials) using MIP/0.2S-B-g-C_3_N_4_/SPR chips, suggesting high sensitivity towards DEOX in comparison with the other studied sensors (Table 1). Since the S-B-g-C_3_N_4_/SPR nanocomposites were prepared for the development of SPR sensors using thermal polycondensation and microwave synthesis methods, minimal waste was generated during sensor preparation. This proves that an environmentally and human-friendly sensor technique was developed, contributing to the scientific literature. In addition, the short determination time and small sample volume requirements make the SPR method more suitable compared to other sensor techniques. Finally, the developed sensor can rapidly detect mycotoxin contamination in drinking water and fruit juice samples and thus prevent metabolic disorders.

### 3.5. Recovery

Recovery experiments were conducted to demonstrate the high specificity of the prepared SPR sensor using real drinking water and orange juice samples. Small amounts of the drinking water and orange juice samples were prepared for analysis and divided into four equal parts. Increasing concentrations (2.0, 5.0, and 7.0 ng L^−1^) of standard DEOX samples were added to each part except for the first part. Nearly 100% recovery was obtained, proving that the prepared SPR sensor detected DEOX in real drinking water and orange juice samples with high selectivity (Table 2). To prove the reliability of the prepared SPR sensor, the results were compared to those of an independent method in the literature [57] using the Kruskal–Wallis test. The lack of significant differences between the analytical results proved the reliability of the prepared SPR sensor (T_calculated_ > T_tabulated_, *p* > 0.05).

Lastly, the standard addition method was applied for DEOX analysis using drinking water and orange juice samples, and calibration equations y (ΔR) = 0.6797 × (C_DEOX_, ng L^−1^) − 1.0437 and y (ΔR) = 0.6701 × (C_DEOX_, ng L^−1^) − 1.1379 were obtained for the drinking water and orange juice samples, respectively. The closeness of the slopes of the direct calibration method and the standard addition method suggested that MIP/0.2S-B-g-C_3_N_4_/SPR chips had high selectivity towards DEOX in drinking water and orange juice samples containing other co-contaminants and mycotoxins.

### 3.6. Selectivity, Repeatability, Reusability, and Reproducibility of MIP/0.2S-B-g-C_3_N_4_/SPR Chip

**Figure 6:**
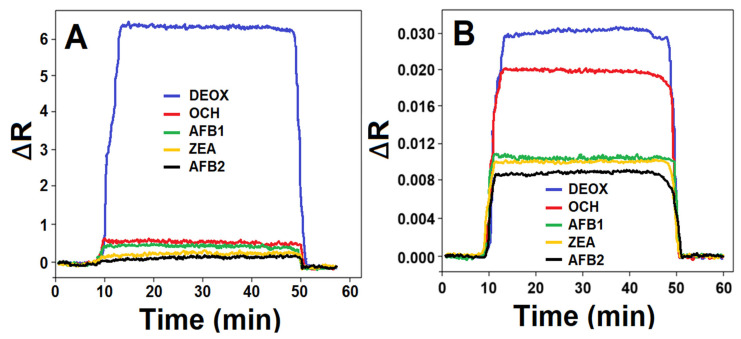
Selectivity of (**A**) MIP/0.2S-B-g-C_3_N_4_/SPR and (**B**) NIP/0.2S-B-g-C_3_N_4_/SPR chips towards 10.0 ng L^−1^ DEOX, 1000.0 ng L^−1^ OCH, 1000.0 ng L^−1^ AFB1, 1000.0 ng L^−1^ ZEA, and 1000.0 ng L^−1^ AFB2.

For the selectivity study of the prepared MIP/0.2S-B-g-C_3_N_4_/SPR chip, OCH, AFB1, ZEA, and AFB2 were selected as competitor agents in addition to DEOX. The SPR sensorgrams obtained by using both the MIP and NIP sensors with different analytes and competitor agents are shown in Figure 6. As expected, the MIP sensor was observed to recognize DEOX 13.00 times more selectively than OCH, 16.25 times more selectively than AFB1, 32.50 times more selectively than ZEA, and 65.00 times more selectively than AFB2. The calculated selectivity coefficient (k) and relative selectivity coefficient (k′) values are given in Table S1. According to Table S1, the sensors produced via molecular imprinting technology to identify DEOX in real drinking water and orange juice samples were proven to have important features such as high selectivity.

To determine the repeatability of the MIP/0.2S-B-g-C_3_N_4_/SPR chip, the produced SPR sensor was utilized for six “adsorption–desorption–regeneration” cycles in the presence of 10.0 ng L^−1^ DEOX. The SPR signal for each cycle was 6.0 ∆R, verifying good repeatability (Figure S6).

The reusability of one MIP/0.2S-B-g-C_3_N_4_/SPR chip was determined: the sensorgrams were constantly monitored in the presence of 10.0 ng L^−1^ DEOX using the proposed MIP/0.2S-B-g-C_3_N_4_/SPR chip for 15 days. The relative standard deviation (RSD) of the monitored signals was 0.09%, verifying high reusability.

For reproducibility experiments, 20 independent MIP/0.2S-B-g-C_3_N_4_/SPR chips were produced, and each SPR chip was tested with 10.0 ng L^−1^ DEOX. The RSD of the calculated SPR signals was 0.37%, suggesting high reproducibility.

### 3.7. Precision and Accuracy of MIP/0.2S-B-g-C_3_N_4_/SPR Sensor

Validation experiments to determine accuracy, intra-day precision, and inter-day precision were completed for three DEOX amounts (2.00, 5.00, and 7.00 ng L^−1^ DEOX) (Table S2). The RSD values were 0.12 for intra-day and 0.12–0.25 for inter-day precision, indicating the high precision of the MIP/0.2S-B-g-C_3_N_4_/SPR sensor.

Accuracy tests were completed by calculating the % relative error (Bias) (Table S2). The low Bias % values of 0.02 to 0.10 were attributed to the high accuracy of the MIP/0.2S-B-g-C_3_N_4_/SPR sensor.

### 3.8. Ruggedness and Robustness of MIP/0.2S-B-g-C_3_N_4_/SPR Sensor

For the ruggedness test, the effects of using two different analysts were examined in the presence of 10.0 ng L^−1^ DEOX on the MIP/0.2S-B-g-C_3_N_4_/SPR sensor, and the obtained results were compared by the Wilcoxon test. According to the Wilcoxon test, no important difference was observed, supporting to the high ruggedness of the proposed sensor (*p* > 0.05). For the robustness test, the effects of small changes in the optimal pH value (pH 6.1 and pH 5.9) on the analysis results were statistically examined. Again, the obtained results were compared by the Wilcoxon test, and no important difference was found, supporting the high robustness of the proposed sensor (*p* > 0.05).

## 4. Conclusions

In summary, a highly reproducible and selective molecularly imprinted SPR sensor based on sulfur-doped boron graphitic carbon nitride was developed to identify deoxynivalenol mycotoxin in drinking water and orange juice samples. The sensor showed linearity from 1.0 to 10.0 ng L^−1^, an LOQ of 1.0 ng L^−1^, and an LOD of 0.30 ng L^−1^, suggesting high sensitivity. Moreover, the SPR sensor detected the mycotoxin deoxynivalenol with satisfactory recoveries. In addition, the prepared nanomaterials were developed with an environmentally friendly technique to produce the SPR sensor. Hence, this superior sensor containing a nanocomposite produced via the molecular imprinting technique has significant potential for usage in environmental and food analysis applications.

## Figures and Tables

**Table 1:** Comparison of MIP/0.2S-B-g-C_3_N_4_/SPR chip with other methods for assaying DEOX.

Material/Method	Linear Range (ng L^−1^)	LOD (ng L^−1^)	Real Sample	Ref.
Cu_2_O nanoparticles	2.0 × 10^3^–1.0 × 10^5^	10.0	Wheat and corn	[51]
Colorimetric immunoassay	1.77 × 10^2^–6.07 × 10^3^	98.0	Corn flour	[52]
Dual-label time-resolved fluoroimmunoassay (TRFIA)	19.40–1.0 × 10^5^	19.4	Cereal samples	[53]
Electrochemiluminescence aptasensor	1.0 × 10^3^–2.0 × 10^7^	300.0	Milk	[54]
Surface enhanced Raman spectroscopy (SERS)	2.96 × 10^2^–2.96 × 10^7^	2.96 × 10^7^	Oats	[55]
HPLC-UV	2.0 × 10^4^–1.0 × 10^6^	4.4 × 10^3^	Baby formula and Korean rice wine	[56]
MIP/0.2S-B-g-C_3_N_4_/SPR	1.0–10.0	0.30	Drinking water and orange juice	This study

**Table 2:** Recovery results of DEOX (*n* = 6).

MIP/0.2S-B-g-C_3_N_4_/SPR				Fluorescence	
Sample	Added DEOX (ng L^−1^)	Found DEOX (ng L^−1^)	* Recovery (%)	Found DEOX (ng L^−1^)	* Recovery (%)
Drinking water	-	1.03 ± 0.01	-	1.02 ± 0.02	-
	2.00	3.06 ± 0.02	100.99 ± 0.01	3.04 ± 0.03	100.66 ± 0.01
	5.00	5.99 ± 0.03	99.34 ± 0.02	6.03 ± 0.01	100.17 ± 0.01
	7.00	8.04 ± 0.02	100.13 ± 0.02	8.02 ± 0.02	100.00 ± 0.02
Orange juice	-	2.02 ± 0.02	-	2.03 ± 0.04	-
	2.00	4.01 ± 0.04	99.75 ± 0.03	4.02 ± 0.03	99.75 ± 0.02
	5.00	7.03 ± 0.04	100.14 ± 0.02	7.02 ± 0.05	99.86 ± 0.01
	7.00	9.03 ± 0.03	100.11 ± 0.01	9.02 ± 0.02	99.89 ± 0.03

* Recovery = found DEOX, ng L^−1^/real DEOX, ng L^−1^.

## Data Availability

The original contributions presented in this study are included in the article/Supplementary Materials. Further inquiries can be directed to the corresponding author.

## Supplementary Materials

The following supporting information can be downloaded at https://www.mdpi.com/article/10.3390/foods15030481/s1, Figure S1: EDX spectrum of 0.2S-B-g-C_3_N_4_ nanocomposite; Figure S2: XRD patterns of (A) g-C_3_N_4_, h-BN, and B-g-C_3_N_4_ and (B) B-g-C_3_N_4_ and S-B-g-C_3_N_4_ nanocomposites; Figure S3: UV–Vis diffuse reflectance spectra of (A) g-C_3_N_4_, h-BN and B-g-C_3_N_4_, and (B) S-B-g-C_3_N_4_ nanocomposites; Figure S4: (A) FTIR spectra of DEOX-imprinted film on 0.2S-B-g-C_3_N_4_/SPR; AFM images of (B) bare SPR chip and (C) DEOX-imprinted film on 0.2S-B-g-C_3_N_4_/SPR sensor; Figure S5: SEM image of (A) MIP/0.2S-B-g-C_3_N_4_/SPR and (B) NIP/0.2S-B-g-C_3_N_4_/SPR; Figure S6: Repeatability of MIP/0.2S-B-g-C_3_N_4_/SPR in the presence of 10.0 ng L^−1^ DEOX in pH 6.0 PB at 25 °C. (a) Adsorption; (b) desorption; (c) regeneration; Table S1: k and k′ values of SPR chips (MIP/0.2S-B-g-C_3_N_4_/SPR and NIP/0.2S-B-g-C_3_N_4_/SPR) (*n* = 6); Table S2: Intra-day and inter-day precision and accuracy results for DEOX (*n* = 6).
