# Supplementary material for: Molecularly Imprinted Deoxynivalenol Surface Plasmon Resonance Sensor Based on Sulfur-Doped Boron Graphitic Carbon Nitride
Source: Foods. 2026 Jan 30;15(3):481. doi: 10.3390/foods15030481 (PMC12896590; doi:10.3390/foods15030481)
Supplement: Supplementary file 1 [file foods-15-00481-s001.zip › foods-4080829-supplementary.pdf]

Supplementary Materials

For

Molecularly Imprinted Deoxynivalenol Surface Plasmon Resonance Sensor Based on Sulfur-Doped Boron Graphitic Carbon Nitride

Müge Mavioğlu Kaya[1], Haci Ahmet Deveci[2], Bahar Bankoğlu Yola[3], İlknur Polat[4], Sena Bekerecioğlu[4], Necip Atar[5], Mehmet Lütfi Yola[6,7*]

[1]Department of Molecular Biology and Genetic, Faculty of Art and Science, Kafkas University, 36000 Kars, Türkiye; m.mavioglu@kafkas.edu.tr (M.M.K)
[2]Department of Nutrition and Dietetics, Faculty of Health Sciences, Gaziantep University, Gaziantep, 27000, Türkiye; h_ahmet_deveci@gantep.edu.tr (H.A.D)
[3]Department of Engineering Basic Sciences, Faculty of Engineering and Natural Sciences, Gaziantep Islam Science and Technology University, Gaziantep, 27260, Türkiye; bahar.bankogluyola@gibtu.edu.tr (B.B.Y)
[4]Department of Nutrition and Dietetics, Faculty of Health Sciences, Hasan Kalyoncu University, Gaziantep, 27010, Türkiye; ilknur.polat@hku.edu.tr (I.P); sena.bekerecioglu@hku.edu.tr (S.B)
[5]Department of Chemical Engineering, Faculty of Engineering, Pamukkale University, Denizli, 20160, Türkiye; natar@pau.edu.tr (N.A)
[6]Department of Biology, Faculty of Science, Ankara University, Ankara, 06100, Türkiye
[7]Integrated Technologies Research Center (BUTAM), Ankara University, Ankara, 06690, Türkiye

*Correspondence: mehmetlutfiyola@ankara.edu.tr (M.L.Y); Tel.: +90-3122168600; Fax: +90-3122868900

2.1. Chemicals and apparatus

Scanning electron microscopy (SEM, ZEISS EVO 50 SEM, Tokyo, Japan), transmission electron microscopy (TEM, JEOL 2100 TEM, Tokyo, Japan), Rigaku X-ray diffractometer (XRD, Germany) and fourier transform infrared spectroscopy (FTIR, Bruker Optics Inc., Ettlingen, Germany) were used for the structural characterizations. Nano magnetics instrument mode atomic force microscopy (AFM, Tokyo, Japan) was used for the observation of surface thicknesses.

3.4. Sensitivity of MIP/0.2S-B-g-C_3N_4/SPR

$$LOQ = 10.0\ S / m$$

$$LOD = 3.3\ S / m$$

S: Standard deviation of the intercept and m: Slope of the regression line

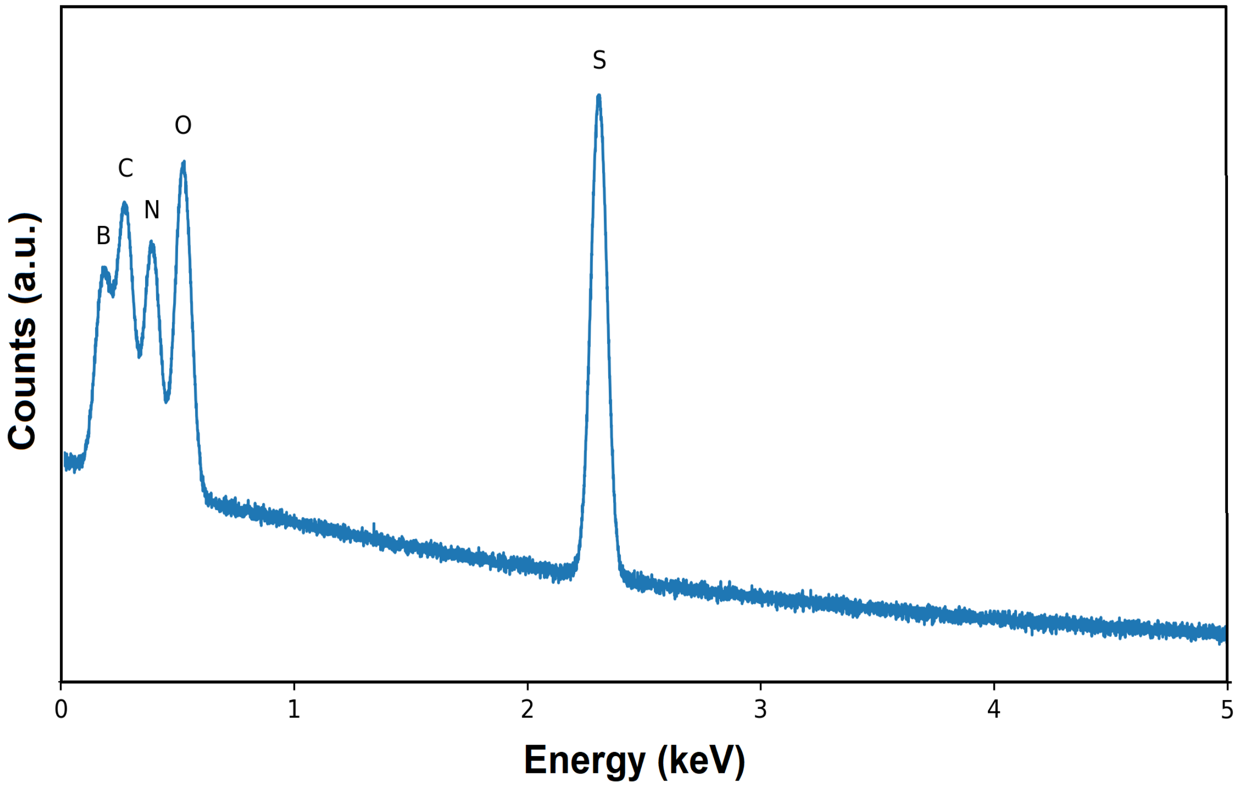

Figure S1. EDX spectrum of 0.2S-B-g-C_3N_4 nanocomposite

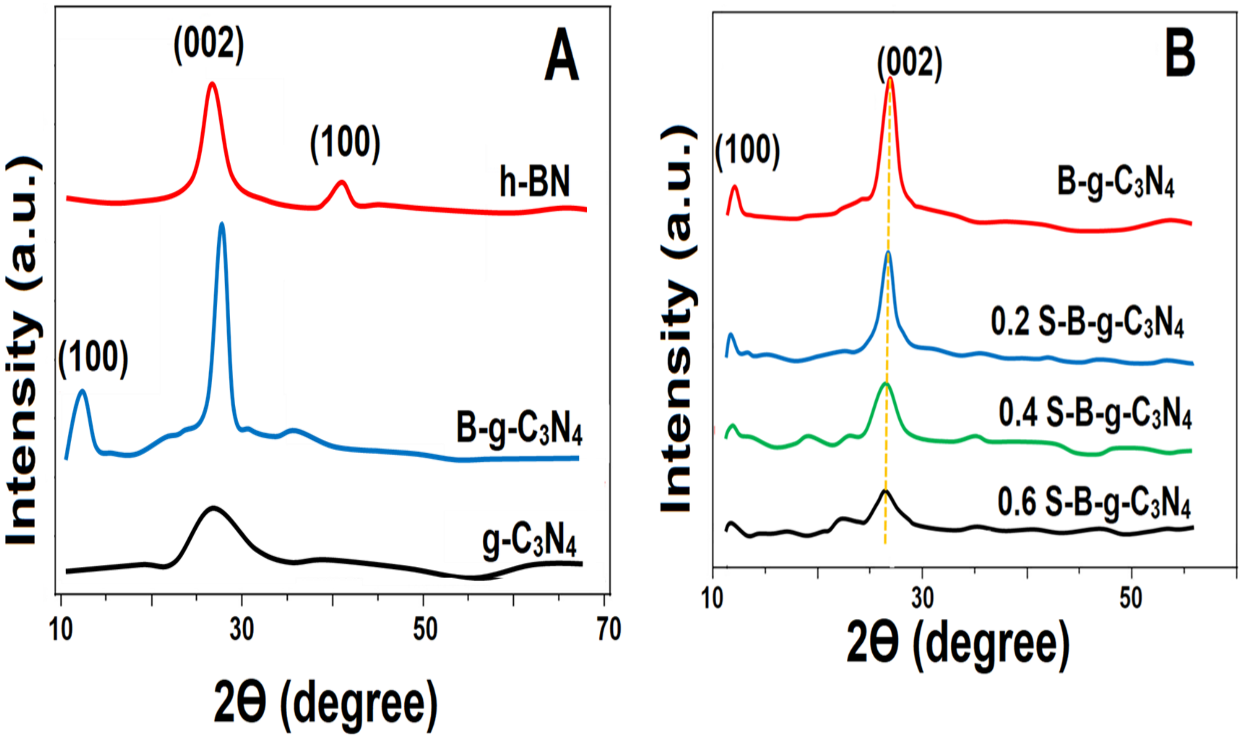

Figure S2. XRD patterns of (A) $g-C_3N_4$, h-BN and $B-g-C_3N_4$ and (B) $B-g-C_3N_4$ and $S-B-g-C_3N_4$ nanocomposites

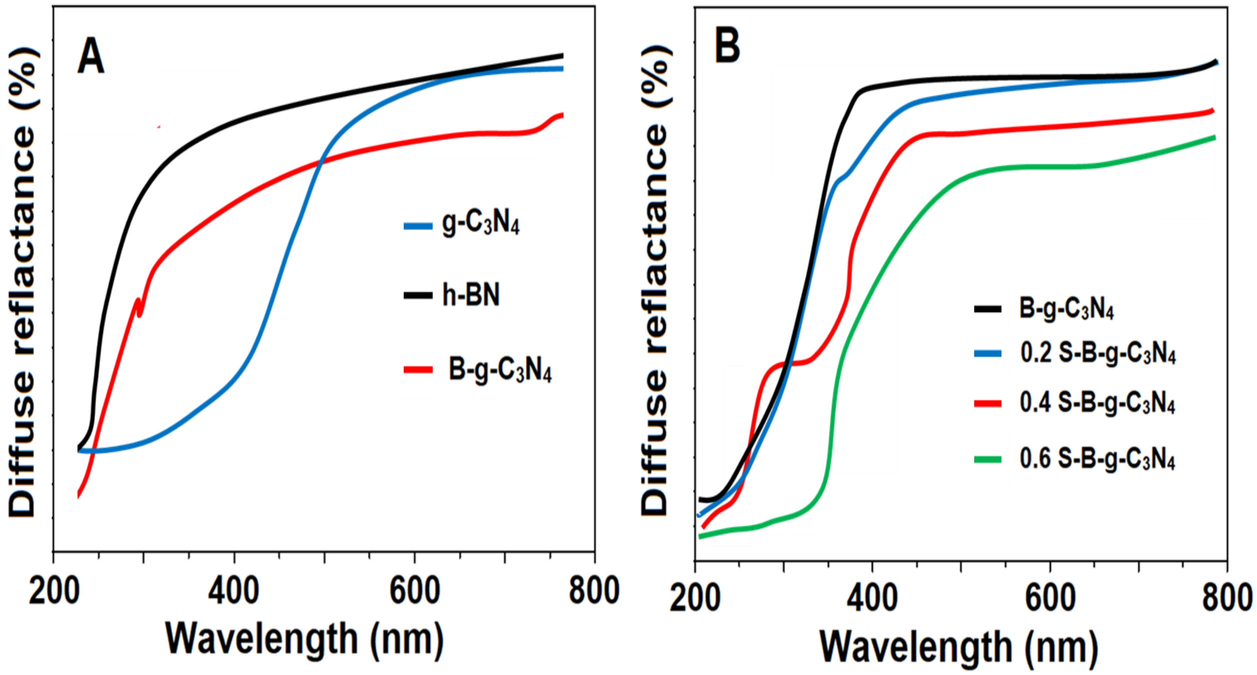

Figure S3. UV–Vis diffuse reflectance spectra of (A) $g-C_3N_4$, h-BN and $B-g-C_3N_4$ and (B) $S-B-g-C_3N_4$ nanocomposites

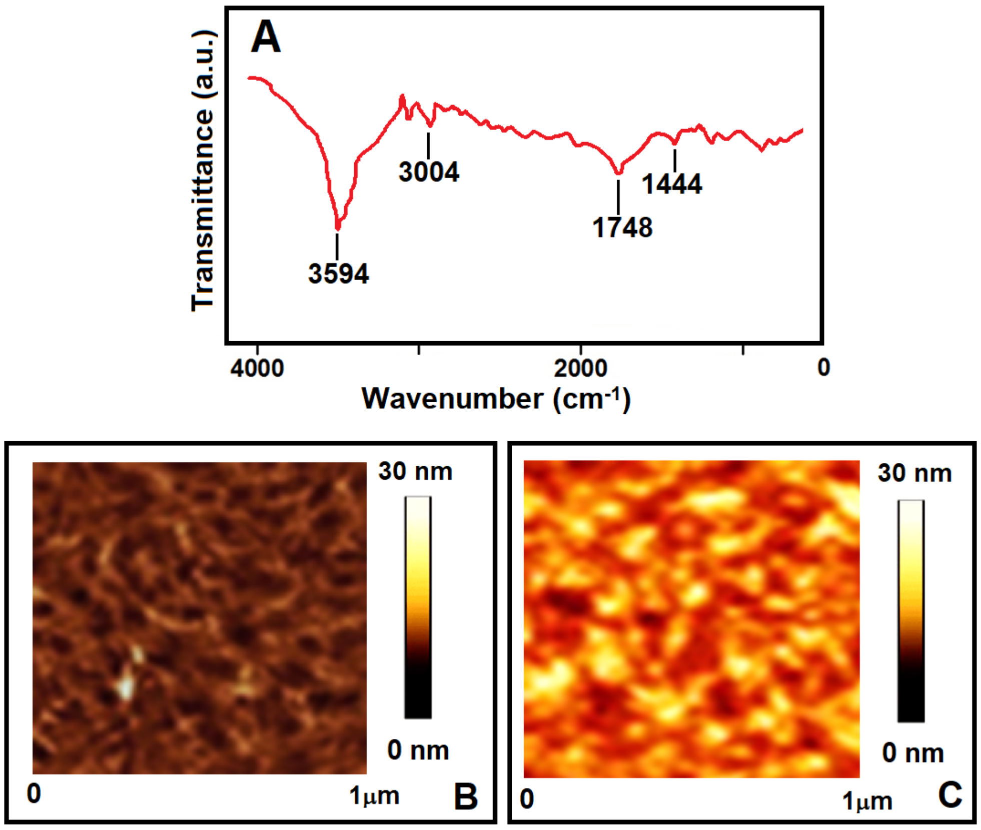

Figure S4. (A) FTIR spectra of DEOX-imprinted film on 0.2S-B-g-C_3N_4/SPR; AFM images of (B) bare SPR chip and (C) DEOX -imprinted film on 0.2S-B-g-C_3N_4/SPR

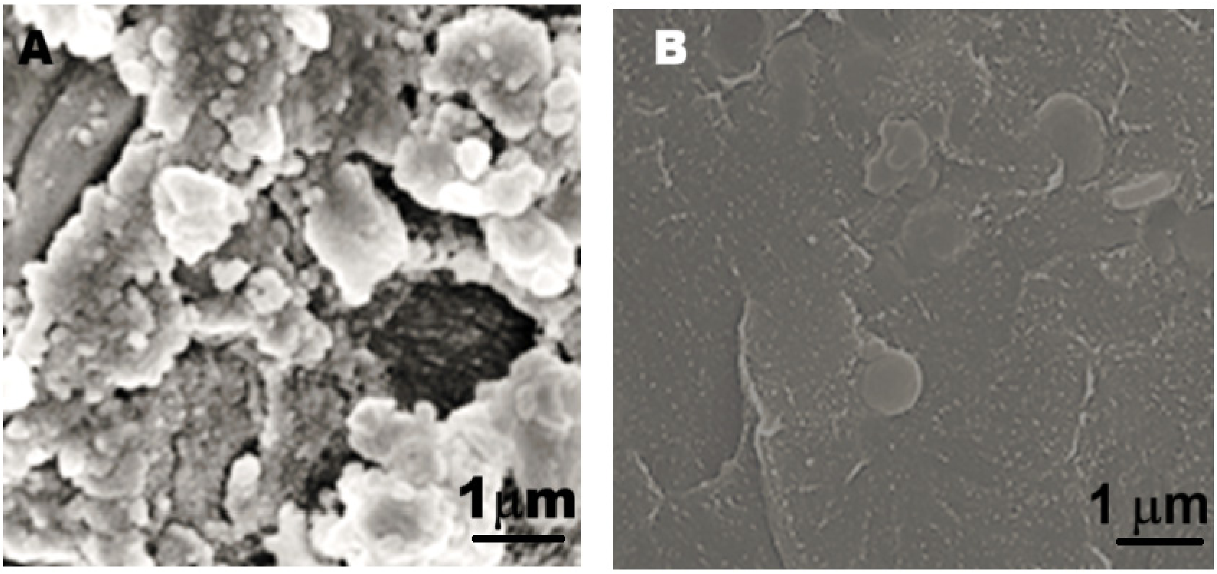

Figure S5. SEM image of (A) MIP/0.2S-B-g-C_3N_4/SPR and (B) NIP/0.2S-B-g-C_3N_4/SPR

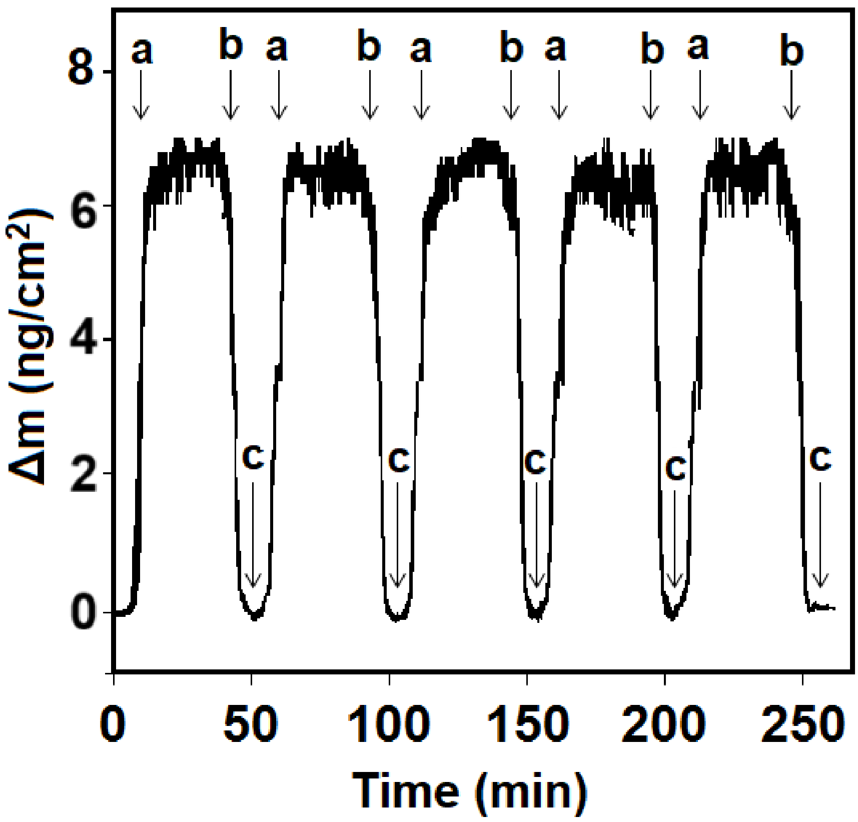

Figure S6. Repeatability of MIP/0.2S-B-g-C_3N_4/SPR in presence of 10.0 ng L^{-1} DEOX including pH 6.0 PB at 25 °C. (a) adsorption; (b) desorption; (c) regeneration

Table S1. k and k′ values of SPR chips (MIP/0.2S-B-g-C_3N_4/SPR and NIP/0.2S-B-g-C_3N_4/SPR) (*n*=6)

	MIP		NIP		
	**ΔR**	**k**	**ΔR**	**k**	**k′**
DEOX	6.50±0.01	-	0.30 ± 0.01	-	-
OCH	0.50±0.03	13.00	0.20 ± 0.02	1.50	8.67
AFB1	0.40±0.01	16.25	0.10± 0.01	3.00	5.42
ZEA	0.20±0.01	32.50	0.10 ± 0.01	3.00	10.83
AFB2	0.10±0.04	65.00	0.05 ± 0.01	6.00	10.83

Analyte concentrations: 10.0 ng L^{-1} DEOX, 1000.0 ng L^{-1} OCH, 1000.0 ng L^{-1} AFB1, 1000.0 ng L^{-1} ZEA, 1000.0 ng L^{-1} AFB2

$k = \Delta R_{DEOX}/\Delta R_{interfering\ chemical}$ and $k' = k_{MIP}/k_{NIP}$.

Table S2. Intra-day and inter-day precision and accuracy results of DEOX (*n*=6)

**Added standard DEOX (ng L^{-1})**	**Intra-day**			**Inter-day**		
	**Found[a] (ng L^{-1})**	**Precision[b] (%)**	**Accuracy[c] (%)**	**Found[a] (ng L^{-1})**	**Precision[b] (%)**	**Accuracy[c] (%)**
2.000	2.002 ± 0.001	0.12	0.10	2.001 ± 0.002	0.25	0.05
4.000	4.000 ± 0.002	0.12	0.03	4.001 ± 0.003	0.18	0.03
6.000	6.001 ± 0.003	0.12	0.02	6.002 ± 0.003	0.12	0.03

[a]Mean ± Standart Error, [b]Precision %: Relative Standart Deviation (RSD), [c]Bias %: [(found – added)/added]×100%
